# Integrated Experimental, Chemometric, and In Silico Analyses of Larvicidal Supercritical Extracts From Myrtaceae Species Against *Aedes aegypti*

**DOI:** 10.1002/cbdv.71786

**Published:** 2026-09-30

**Authors:** Ubiratan Alegransi Bones, Regiane Chiamente Pessetti, Gilberto Diniz Cozzer, Iuri Raiel Giacomelli, Maike Valentim Buzatto, Walter Antônio Roman Junior, Renan de Souza Rezende, Jacir Dal Magro, Vanessa da Silva Corralo

**Affiliations:** ^1^ Postgraduate Program in Health Sciences Community University of the Chapecó Region – UNO Chapecó Santa Catarina Brazil; ^2^ Postgraduate Program in Environmental Sciences Community University of the Chapecó Region – UNO Chapecó Santa Catarina Brazil

**Keywords:** arboviruses 1, dengue 2, supercritical extract 3, vector control 4

## Abstract

Supercritical extracts from five Myrtaceae species were evaluated for larvicidal activity against *Aedes aegypti* (*A. aegypti*) and investigated through chemical profiling, concentration‐response modeling, chemometric analysis, and molecular modeling. Leaf extracts of *Corymbia citriodora, Eugenia uniflora* (*E. uniflora*)*, Plinia cauliflora* (*P. cauliflora*)*, Psidium cattleianum* (*P. cattleianum*), and *Psidium guajava* were characterized by GC–MS and tested at 60–300 µg mL^−^^1^ for 72 h. We also performed molecular docking analyses of the identified compounds against two *A. aegypti* targets, AeSCP‐2 (PDB: 1PZ4) and AeHKT (PDB: 6MFB), and *Drosophila melanogaster* acetylcholinesterase (DmAChE; PDB: 6XYU), a homologous model of insect AChE. All extracts caused mortality, although responses differed among species. *P. cattleianum* and *E. uniflora* were highly active across the tested range, whereas *P. cauliflora* showed weaker, heterogeneous, and non‐monotonic responses. PCA revealed marked compositional differences among species but no significant mortality gradient along the first two components. A grouped cross‐validated regression tree showed modest predictive performance, indicating associations between covarying chemical profiles and mortality rather than independent compound effects. Docking against AeSCP‐2, AeHKT, and DmAChE, followed by molecular dynamics of the AeSCP‐2–β‐sitosterol complex, identified structurally plausible ligand‐target associations. Overall, the study demonstrates species‐dependent larvicidal activity and provides experimentally testable hypotheses for prioritizing extracts, compounds, and molecular targets in future investigations.

## Graphical abstract

The cross‐validated regression tree showed modest predictive performance. Mortality was not significantly associated with the first two PCA axes. Molecular modeling generated testable ligand–target hypotheses. *Psidium cattleianum* had lower LD50. The larvicidal activity of the crude extracts could not be attributed to individual constituents.

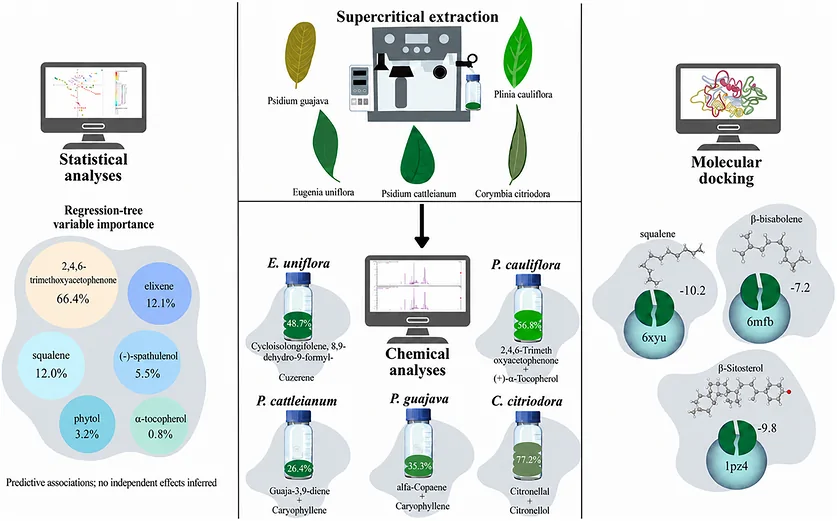

## 1 Introduction

Vector‐borne diseases represent a major contemporary public health challenge, accounting for a substantial fraction of the global burden of infectious diseases and affecting hundreds of millions of people, mainly in low‐ and middle‐income countries [1]. In the Americas alone, a total of 3 141 351 arboviral cases were reported in 2022. Of these, 2 826 982 (90.0%) were dengue cases, 273 841 (8.7%) were chikungunya cases, and 40 528 (1.3%) were zika cases [2].

Among these diseases, dengue is one of the most important arboviral diseases worldwide. Billions of people live in at risk areas and that of the tens of millions of symptomatic infections occur, resulting in approximately 40 000 deaths per year [3, 4, 5]. This epidemiological scenario is strongly driven by the wide distribution of *Aedes aegypti* (*A. aegypti*), a vector highly adapted to urban and peri‐urban environments [6, 7].

Given the still limited effectiveness of clinical strategies in reducing population‐level transmission of these arboviruses, vector control remains one of the pillars of dengue management and other Aedes‐borne diseases [8]. Historically, this control has relied on the intensive use of chemical insecticides, especially organophosphates and pyrethroids, as well as growth regulators [9, 10]. However, the continued use of these compounds promotes the selection of resistant populations and increases ecotoxicological concerns related to environmental persistence and effects on nontarget organisms [9, 11, 12].

In parallel, the search for new insecticidal agents has intensified, with natural compounds considered a promising source. Owing to their structural diversity, these substances can act through alternative mechanisms and modulate physiological pathways essential for larval development [13]. In addition, the identification and characterization of these molecules have been facilitated by advanced extraction techniques, such as supercritical carbon dioxide extraction, which yield more selective and stable extracts that better represent the original chemical composition of the plant material while preserving the biological activity of volatile and thermolabile constituents [14].

Additionally, in silico approaches, such as molecular docking, have been widely used to predict interactions between bioactive metabolites and proteins involved in the survival and development of *A. aegypti*. These approaches complement in vivo assays by enabling the investigation of individual ligand–target associations [15, 16, 17].

In this study, we investigated the larvicidal potential of supercritical leaf extracts obtained from five Myrtaceae species (*Eugenia uniflora, Plinia cauliflora*, *Corymbia citriodora*, *Psidium guajava*, and *Psidium cattleianum*) against *A. aegypti* larvae. By integrating experimental bioassays, GC–MS‐based chemical characterization, chemometric analyses, and molecular modeling, we evaluated species‐ and concentration‐related patterns of larval mortality in relation to extract chemical profiles and generated candidate ligand–target hypotheses for subsequent experimental testing.

## 2 Results

### 2.1 Chemical Characterization

The chemical characterization of the extracts revealed a diverse profile of secondary metabolites. In the set of five species investigated, 71 phytochemicals were putatively identified, spanning different structural classes. Among the species studied, the highest extract yield was obtained from *Corymbia citriodora* (*C. citriodora*) (4.57% ± 0.28), followed by *E. uniflora* (3.08% ± 0.016), *P. cattleianum* (2.38% ± 0.032), *P. guajava* (1.87% ± 0.047), and *P. cauliflora* (1.52% ± 0.01). This broad chemical profile highlights the metabolic richness of the analyzed species and provides a robust basis for investigating their biological properties. In the species *E. uniflora*, curzerene (27.16%) and cycloisolongifolene 8,9‐dehydro‐9‐formyl (21.45%) predominated. In *P. cauliflora*, the major compound was 2,4,6‐trimethoxyacetophenone (44.54%), with a substantial contribution from (±)‐α‐tocopherol (12.22%). In *P. guajava*, caryophyllene (18.41%), α‐copaene (16.90%), and humulene (14.35%) were prominent, in addition to 4,5‐di‐epi‐aristolochene (9.13%). In *C. citriodora*, the composition was dominated by citronellal (55.41%) and citronellol (21.79%). In *P. cattleianum*, the main constituents were caryophyllene (17.18 %), guaia‐3,9‐diene (9.16%), β‐cadinene (7.46%), and α‐selinene (7.12%) (Table 1).

**TABLE 1:** Chemical constituents of the supercritical extracts of the species *Eugenia uniflora, Plinia cauliflora, Psidium guajava, Corymbia citriodora, and Psidium cattleianum*, putatively identified by gas chromatography coupled to mass spectrometry.

*Eugenia uniflora (%)*		*RT*	*Plinia cauliflora (%)*		*RT*	*Psidium guajava (%)*		*RT*	*Corymbia citriodora (%)*		*RT*	*Psidium cattleianum (%)*		*RT*
γ‐Elemene	3.32	9.974	Isocaryophyllene	2.34	10.392	alpha.‐Copaene	16.89	9.793	D‐Limonene	0.4	4.978	α‐Terpineol	0.95	7.221
β‐Elemene	2.71	12.671	Germacrene D	1.21	11.159	Caryophyllene	18.41	10.395	Isopulegol	2.75	6.603	alpha.‐Copaene	1.71	9.793
Isocaryophyllene	1.47	10.499	α‐Selinene	0.73	11.235	Humulene	14.35	10.823	Citronellal	55.41	6.655	Caryophyllene	17.18	10.395
(‐)‐γ‐Elemene	0.90	10.921	Chamigren	1.46	11.339	4,5‐di‐epi‐aristolochene	9.13	11.012	Citronellol	21.79	7.661	Humulene	1.81	10.823
Alloaromadendrene	0.65	11.159	(‐)‐Spathulenol	4.16	12.335	2‐Isopropenyl‐4a,8‐dimethyl‐1,2,3,4,4a,5,6,7‐octahydronaphthalene	5.62	11.064	(R)‐(+)‐Citronellic acid	1.35	8.739	γ‐Muurolene	3.83	11.067
β‐Cubebene	9.65	11.315	Caryophyllene oxide	2.44	12.424	β‐Eudesmene	6.62	11.238	Cyclohexanol, 2‐(2‐hydroxy‐2‐propyl)‐5‐methyl‐	3.59	9.164	β‐Selinene	5.61	11.235
Curzerene	27.16	11.351	Viridiflorol	0.49	12.525	α‐Selinene	6.05	11.339	Citronellol acetate	0.87	9.341	α‐Selinene	7.12	11.336
Elixene	2.83	11.559	2,4,6‐Trimethoxyacetophenone	44.54	14.474	β‐Cadinene	3.45	11.626	Caryophyllene	5.12	10.392	β‐Bisabolene	1.28	11.4
Cubedol	1.38	11.626	Phytol	3.47	17.712	E‐Nerolidol	3.03	12.024	β‐Cubebene	0.68	11.159	γ‐Cadinene	1.87	11.541
β‐Cadinene	0.51	12.335	5‐(7a‐Isopropenyl‐4,5‐dimethyl‐octahydroinden‐4‐yl)‐3‐methyl‐penta‐2,4‐dien‐1‐ol	5.22	20.047	Caryophyllene oxide	3.24	12.427	Elixene	1.14	11.348	β‐Cadinene	7.46	11.629
(‐)‐Spathulenol	6.61	12.671	Butyl 9,12,15‐octadecatrienoate	1.09	20.844	Juniper camphor	3.66	12.754	Tetraprenol	6.89	18.484	Guaia‐3,9‐diene	9.16	11.825
Isoshyobunone	0.77	12.946	(±)‐α‐Tocopherol	12.22	22.271	Isoaromadendrene epoxide	1.65	12.904				3,7(11)‐Selinadiene	4.48	11.907
Ledene oxide‐(II)	0.5	13.01	β‐Amyrin	2.36	22.488	Tetracyclo[6.3.2.0(2,5).0(1,8)]tridecan‐9‐ol, 4,4‐dimethyl‐	2.68	12.977				E‐Nerolidol	1.3	12.024
Germacrone	3.17	13.71	Dioctyl isophthalate	0.7	22.851	Torreyol	0.97	13.096				γ‐Elemene	2.06	12.109
cycloisolongifolene 8,9‐dehydro‐9‐formyl	21.45	14.013	Squalene	6.54	23.438	Longipinocarveol, trans‐	0.58	13.512				Caryophyllene oxide	1.35	12.424
2‐(7‐Hydroxymethyl‐3,11‐dimethyl‐dodeca‐2,6,10‐trienyl)‐[1,4]benzoquinone	1.06	15.241				Phytol	0.46	17.713				Guaiol	0.41	12.531
Bicyclo[5.1.0]octan‐2‐one, 4,6‐diisopropylidene‐8,8‐dimethyl‐	4.25	15.394				β‐Sitosterol	1.04	20.053				Cubenol	0.55	12.903
5‐Benzofuranacetic acid, 6‐ethenyl‐2,4,5,6,7,7a‐hexahydro‐3,6‐dimethyl‐α‐methylene‐2‐oxo‐, methyl ester	1.29	15.455				dl‐α‐Tocopherol	0.76	22.262				Guaiene	0.55	12.94
Androstan‐17‐one, 3‐ethyl‐3‐hydroxy‐, (5α)‐	2.15	15.601				Squalene	0.9	23.441				Muurolol T	1.5	13.05
Phytol	0.77	17.713										Torreyol	5.34	13.093
(±)‐α‐Tocopherol	1.98	22.262										α‐Eudesmol	3.06	13.206
α‐Amyrin	2.1	22.467										γ‐Eudesmol	2.7	13.365
Squalene	2.35	23.438										Eudesm‐7(11)‐en‐4‐ol	1.56	13.704
Heptacosane	1.02	23.863										4,4,8‐Trimethyltricyclo[6.3.1.0(1,5)]dodecane‐2,9‐diol	0.76	15.614
												Aspidinol	2.69	18.733
												5‐(7a‐Isopropenyl‐4,5‐dimethyl‐octahydroinden‐4‐yl)‐3‐methyl‐penta‐2,4‐dien‐1‐ol	2.59	20.044
												(±)‐α‐Tocopherol	5.56	22.265
												Squalene	3.95	23.438

^*^ *Note*: Species names followed by (%) identify the extract‐specific columns; values indicate the relative abundance of each detected constituent.

Abbreviation: RT, retention time.

### 2.2 Larvicidal Activity and Concentration‐Response Patterns

No mortality occurred in the 0.4% acetone control during the 72‐h observation period (six microcosms; 90 larvae). The positive control confirmed the responsiveness of the bioassay system. Bti exhibited a concentration‐dependent larvicidal effect, with an estimated LC_50_ of 0.0916 mg L^−^^1^ (95% CI: 0.0713–0.117 mg L^−^^1^). By contrast, all five extracts produced measurable larvicidal activity, although the magnitude and concentration dependence differed markedly among species (Figure 1 and Tables S1–S3). *Psidium cattleianum* was the most active extract across the tested range, producing 92.2 +/−9.8% mortality at 60 µg mL^−^^1^ and 100% mortality at 240–300 µg mL^−^^1^. *Eugenia uniflora* caused 83.3 +/−11.7% mortality at 60 µg mL^−^^1^ and complete mortality from 120 µg mL^−^^1^ onward. Mortality following *Psidium guajava* (*P. guajava*) exposure increased from 72.2 +/−9.8% at 60 µg mL^−^^1^ to 100% at 300 µg mL^−^^1^. *Corymbia citriodora* showed a steep concentration response, rising from 52.2 +/−13.6% at 60 µg mL^−^^1^ to 98.9 +/−2.7% at 180 µg mL^−^^1^ and 100% at 240–300 µg mL^−^^1^. *Plinia cauliflora* was less active and substantially more variable, with mean mortality ranging from 31.1% to 64.4% across concentrations.

**FIGURE 1:**
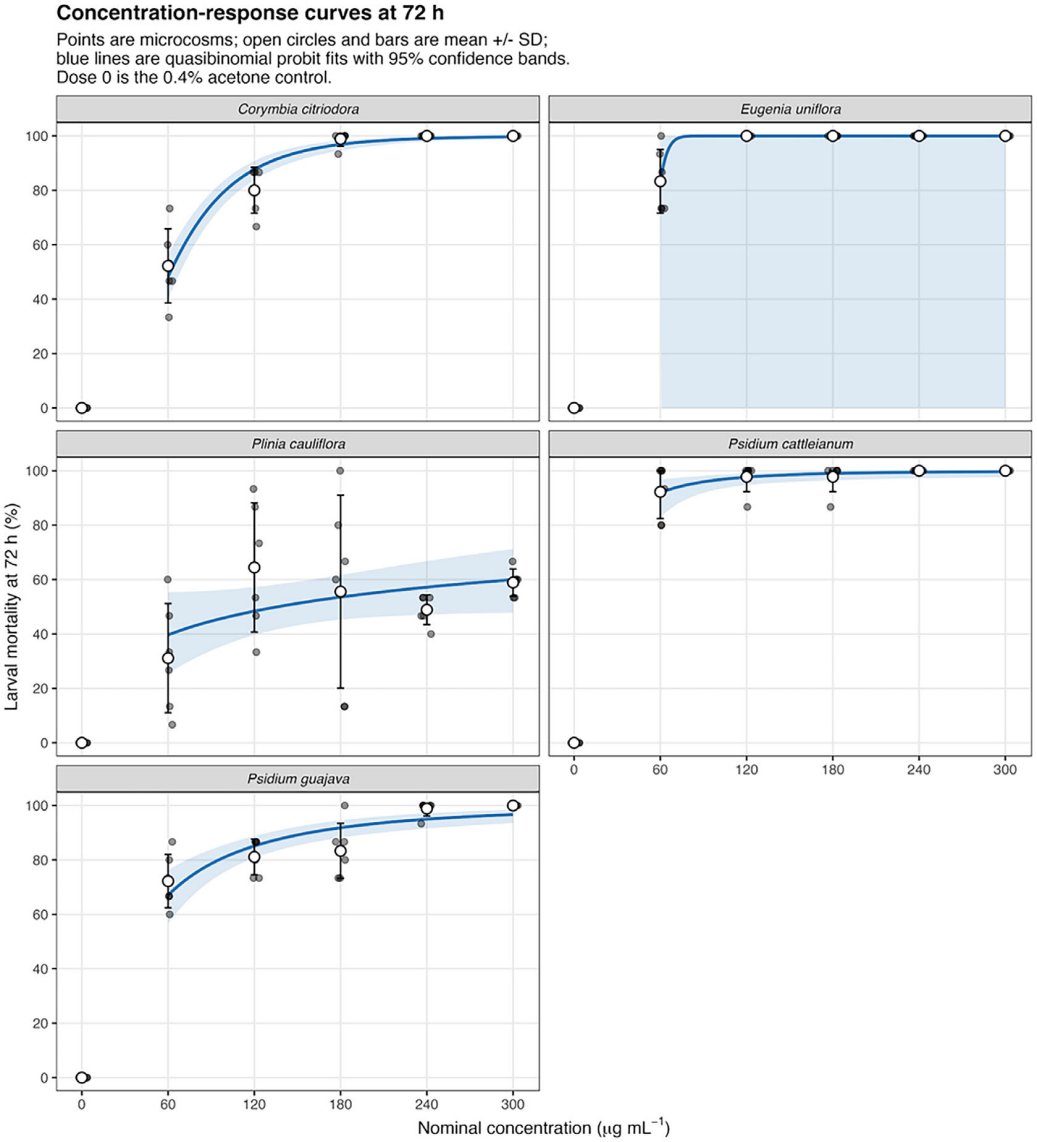
Concentration‐response relationships for cumulative mortality of *Aedes aegypti* larvae after 72 h of exposure to Myrtaceae extracts. Small filled points represent mortality in individual microcosms (15 larvae per microcosm; six independent microcosms per treatment). Open circles and error bars represent the mean ± SD. Blue lines show quasibinomial probit models fitted as a function of log10‐transformed extract concentration, and shaded areas indicate the corresponding 95% confidence bands. The models were fitted using the five positive concentrations (60, 120, 180, 240, and 300 µg mL^−^^1^). A concentration of 0 represents the solvent control containing 0.4% acetone and no extract; control observations are displayed for reference but were not included in the log‐concentration models. Panels correspond to *Corymbia citriodora*, *Eugenia uniflora*, *Plinia cauliflora*, *Psidium cattleianum*, and *Psidium guajava*.

The temporal trajectories corroborated these differences (Figure S1). By 24 h, mortality had already reached 92.2% for *E. uniflora* at 120 µg mL^−^^1^ and at least 94.4% for *P. cattleianum* at 180–240 µg mL^−^^1^, whereas lower concentrations of *C. citriodora* and *P. guajava* accumulated mortality more gradually between 24 and 72 h. *P. cauliflora* showed both slower mortality accumulation and pronounced among‐microcosm heterogeneity.

### 2.3 LC_50_ Estimates and Limitations of Interpretation

Species‐specific quasibinomial probit models yielded 72‐h LC_50_ estimates of 11.57 µg mL^−^^1^ for *P. cattleianum* (95% CI: 2.31–58.03), 36.10 µg mL^−^^1^ for *P. guajava* (22.72–57.36), 52.87 µg mL^−^^1^ for *E. uniflora* (approximately 8.2 x 10‐7 to 3.43 x 109), 61.58 µg mL^−^^1^ for *C. citriodora* (54.73–69.28), and 136.18 µg mL^−^^1^ for *P. cauliflora* (71.17–260.57; Table S4). The degree of empirical support differed among these estimates. Mortality already exceeded 50% at the lowest tested concentration for *P. cattleianum, P. guajava, E. uniflora*, and slightly for *C. citriodora*; their LC50 values were therefore estimated below or near the lower experimental boundary rather than bracketed by observations on both sides of 50%. The *E. uniflora* interval was effectively uninformative because mortality was already high at 60 µg mL^−^^1^ and reached 100% at every higher concentration. The *P. cauliflora* estimate fell within the tested range but was weakened by extra‐binomial dispersion (2.996), a shallow probit slope (0.737), and a non‐monotonic mean response (Spearman rho = 0.30). In contrast, the *C. citriodora* estimate was comparatively precise, although its point estimate remained close to the lower concentration boundary.

### 2.4 Chemical Composition was Strongly Structured by Species and Concentration

The PCA of the 26 treatment centroids resolved pronounced compositional structure (Figure 2). PC1 accounted for 32.85% of the standardized chemical variance and PC2 for 25.50%, jointly explaining 58.35%; inclusion of PC3 increased cumulative explained variance to 78.62%. Only the first five components had non‐negligible eigenvalues and together accounted for effectively all variance, reflecting the highly constrained covariance structure generated by five species‐specific concentration series. Along PC1, positive loadings were greatest for alpha‐selinene, torreyol, beta‐cadinene, and caryophyllene, whereas elixene, β‐cubebene, (‐)‐gamma‐elemene, and several co‐occurring compounds loaded negatively. The largest positive PC2 loading was observed for γ‐elemene and a suite including aspidinol, guaia‐3,9‐diene, eudesm‐7(11)‐en‐4‐ol, gamma‐eudesmol, beta‐selinene, gamma‐muurolene, guaiol, and alpha‐terpineol; β‐sitosterol had the strongest negative PC2 loading. Because many compounds shared almost identical loadings, these axes represent covarying chemical assemblages rather than separable effects of individual constituents.

**FIGURE 2:**
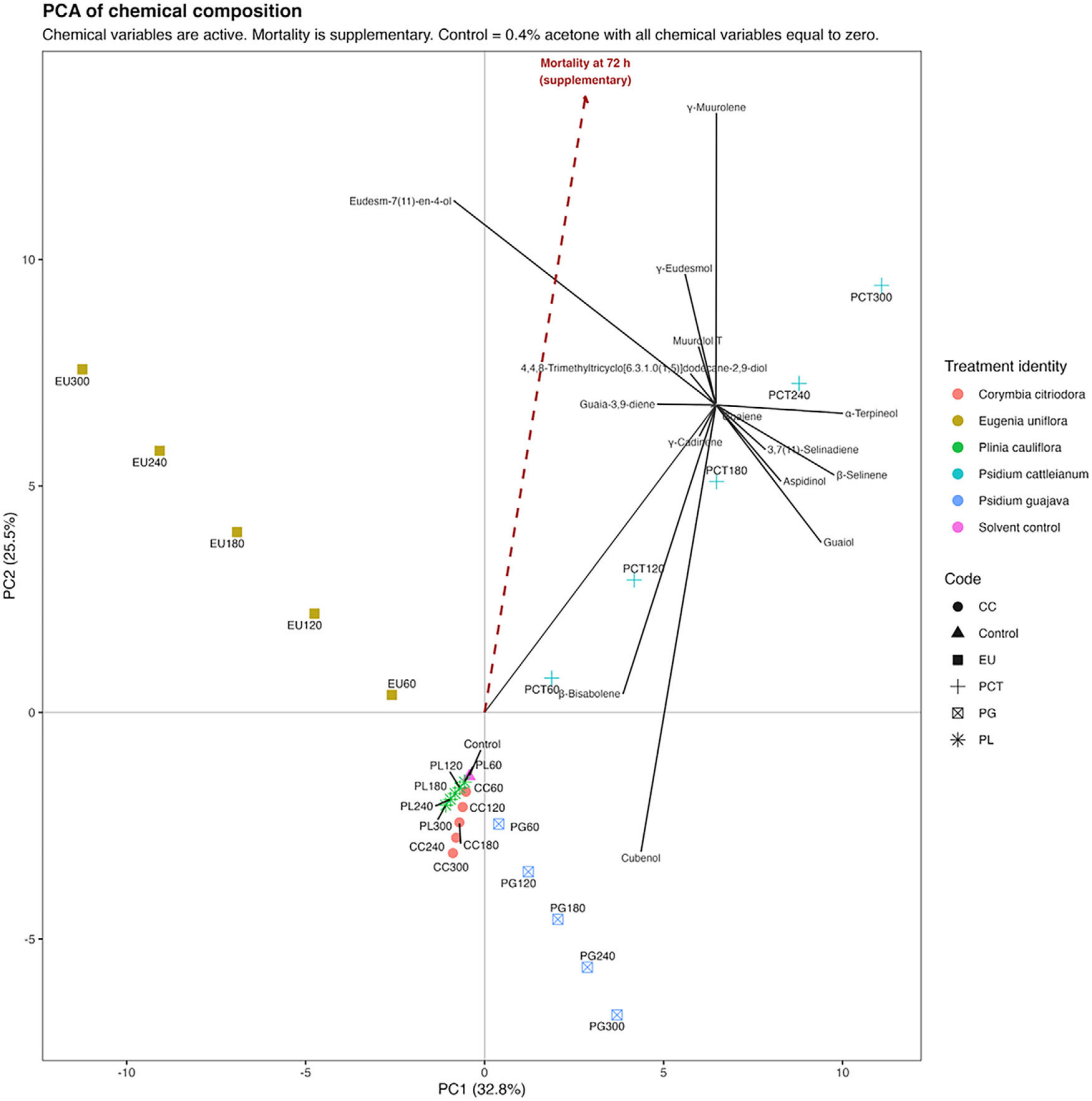
Principal component analysis of the chemical composition of Myrtaceae extracts. The PCA was fitted using 71 nonconstant chemical variables measured across 26 treatment centroids, comprising 25 species‐by‐concentration combinations and one solvent‐control centroid. Chemical variables were centered and scaled before analysis. PC1 and PC2 explained 32.85% and 25.50% of the total chemical variance, respectively, accounting jointly for 58.35%. Points represent treatment centroids and are identified by plant species and nominal concentration; the solvent control contained 0.4% acetone and no extract, and its chemical variables were set to zero. Black arrows show the 15 chemical variables with the largest loading‐vector magnitudes; arrow direction and length indicate their association with the displayed components after common graphical scaling. Mortality at 72 h was excluded from PCA construction and subsequently projected as a supplementary variable using Pearson correlations with PC1 and PC2 (red dashed arrow). Consequently, the mortality vector does not contribute to axis construction and should be interpreted as a descriptive association rather than evidence of causation, synergy, antagonism, or a causal chemical threshold.

Treatment scores separated the five species and traced concentration‐dependent trajectories within species. *P. cattleianum* moved strongly towards positive PC1 and PC2 scores as concentration increased, whereas *E. uniflora* moved toward increasingly negative PC1 and positive PC2 scores. *P. guajava* shifted towards positive PC1 and negative PC2, while *C. citriodora* and *P. cauliflora* occupied a narrower negative‐PC1/negative‐PC2 region. The solvent‐control centroid was located near the latter treatments in the PC1‐PC2 projection; its inclusion reflects a chemically null reference, not a biological extract profile.

Mortality, projected only after the PCA was fitted, was weakly associated with PC1 (*r* = 0.066, *p* = 0.749) and showed a moderate but nonsignificant association with PC2 (*r* = 0.319, *p* = 0.113). Thus, the two‐dimensional ordination robustly described chemical differentiation among extracts but did not support a dominant mortality gradient along either displayed axis. The biplot should therefore be interpreted as a map of compositional variation with a supplementary mortality overlay, not as evidence that any loading caused toxicity.

### 2.5 Regression‐Tree Predictive Performance was Modest

The cost‐complexity‐pruned regression tree was evaluated using 100 repeated grouped fivefold cross‐validations. The minimum mean validation RMSE occurred for the unpruned candidate (CP = 0; RMSE = 20.99 percentage points), but the one‐standard‐error rule selected a simpler tree with CP = 0.002317 because its RMSE remained below the one‐SE limit of 21.17 percentage points (Table S5). For this final tree, mean cross‐validated R‐squared was 0.125 (SD = 0.155; 95% percentile interval: ‐0.287 to 0.388), RMSE was 21.07 percentage points (SD = 1.85; interval: 17.69–25.66), and MAE was 12.38 percentage points (SD = 1.23; Table S6 and Figure 3). The broad R‐squared interval, including negative values in some partitions, indicates variable and generally modest predictive performance for extract‐concentration treatments not represented during model fitting.

**FIGURE 3:**
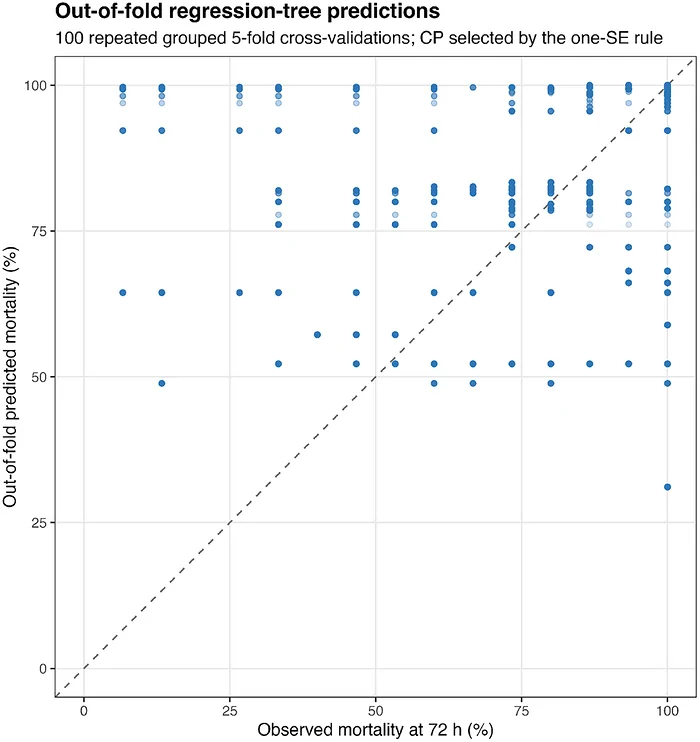
Observed versus cross‐validated predictions of *Aedes aegypti* larval mortality at 72 h. Predictions were generated using 100 repetitions of grouped fivefold cross‐validation of the pruned regression tree. Each microcosm contributed one held‐out prediction per cross‐validation repetition, and all six microcosms from a given species‐by‐concentration treatment were assigned to the same fold. Points represent observed and out‐of‐fold predicted mortality values; transparency was used to reveal overlapping observations. The dashed diagonal line represents perfect agreement between observed and predicted values. Cross‐validated performance was *R* ^2^ = 0.125, RMSE = 21.07 percentage points, and MAE = 12.38 percentage points. These values quantify predictive performance on held‐out treatments and not the goodness of fit of the final tree to the complete dataset.

Within the final tree, 2,4,6‐trimethoxyacetophenone accounted for 66.4% of the total variable‐importance score, followed by elixene (12.1%), squalene (12.0%), (‐)‐spathulenol (5.5%), phytol (3.2%), and alpha‐tocopherol (0.8%; Figure 4). These importance values identify compounds used by the tree to partition the observed multivariate chemical profiles, but they do not isolate independent effects because constituents covaried strongly across crude extracts. Accordingly, neither variable importance nor node split points establish physiological thresholds, synergy, antagonism, or causal effects of individual compounds.

**FIGURE 4:**
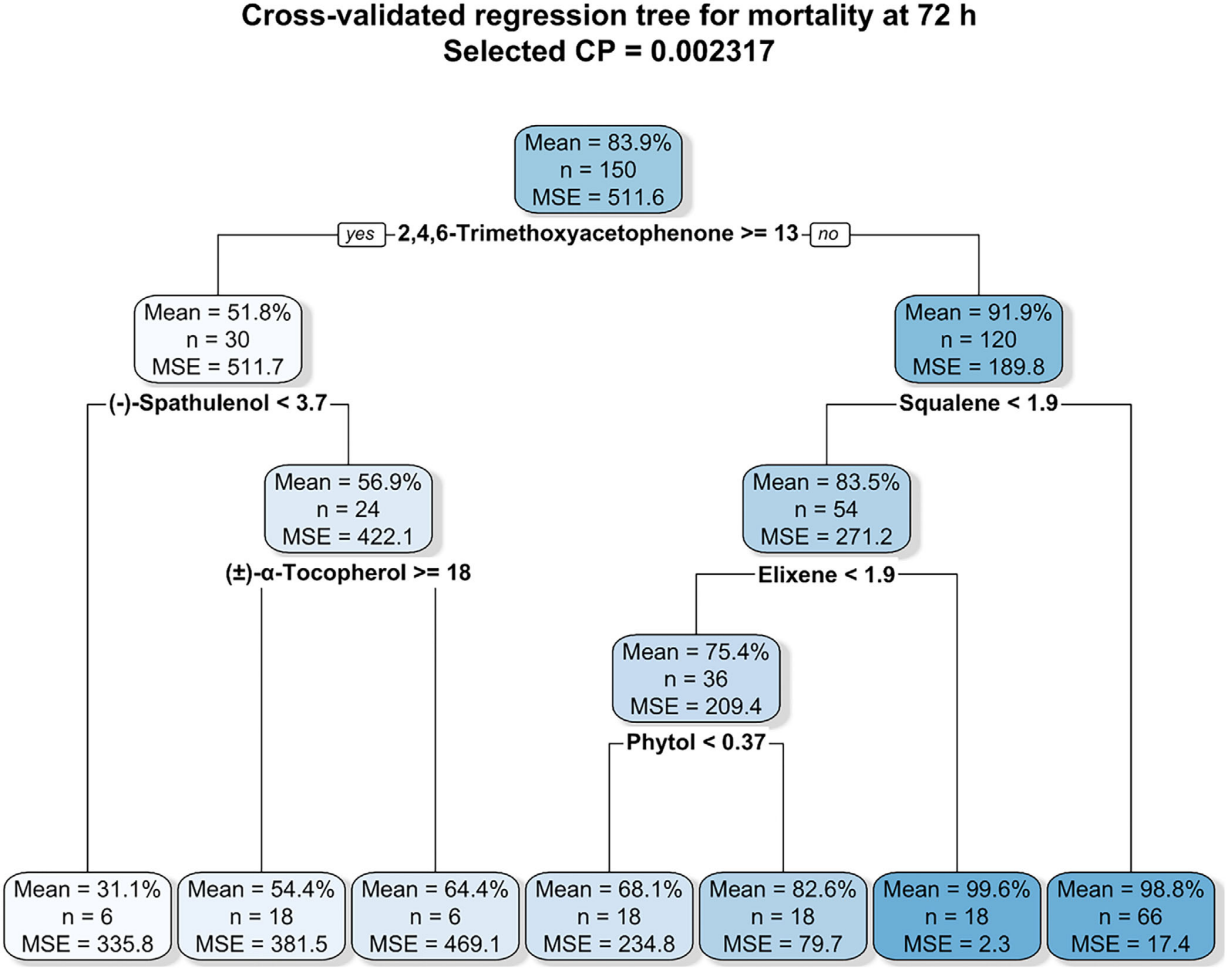
Cross‐validated regression tree for mortality of *Aedes aegypti* larvae after 72 h of exposure to Myrtaceae extracts. The final regression tree was fitted to all 150 extract‐exposed microcosms after model complexity was selected using 100 repetitions of grouped fivefold cross‐validation. The six microcosms belonging to the same species‐by‐concentration treatment were retained in the same fold to prevent information leakage. Internal‐node labels indicate the chemical variable and split value used to partition the observations; terminal‐node labels report mean mortality, number of microcosms (*n*), and within‐node mean squared error. The solvent control was excluded because it did not represent an extract chemical profile. Tree splits identify predictive partitions within the observed dataset and must not be interpreted as causal chemical thresholds or evidence of synergistic or antagonistic interactions.

### 2.6 Molecular Docking Analyses

Acetylcholinesterase (AChE), sterol carrier protein‐2 (SCP‐2), and 3‐hydroxykynurenine transaminase (HKT) were selected based on their established insecticidal relevance in *A. aegypti*. AChE is targeted by conventional organophosphate and carbamate insecticides, and its inhibition disrupts cholinergic neurotransmission. Terpenoid constituents have previously been evaluated against AChE in larvicidal docking studies [18]. SCP‐2 mediates the intracellular transport of dietary cholesterol in the mosquito larval midgut, supporting membrane biogenesis and ecdysteroid synthesis. Because mosquitoes cannot synthesize sterols de novo, AeSCP‐2 has been investigated as an insecticidal target [19, 20], and its pairing with AChE has direct precedent in terpenoid docking studies [18]. HKT is a mosquito‐specific detoxification enzyme that converts the neurotoxic intermediate 3‐hydroxykynurenine into xanthurenic acid; its inhibition is expected to cause oxidative damage from 3‐HK accumulation, and it has been proposed as a selective larvicidal target because of its crystallographically resolved, mosquito‐exclusive active site [15, 21].

Among the constituents detected in the *P. cattleianum* extract, squalene showed a high interaction for the sterol‐2 carrier protein of *A. aegypti* (PDB: 1PZ4), with binding energy of −9.4 kcal/mol, indicating a favorable predicted docking score for this target. In the same extract, the β‐bisabolene showed the best interaction with 3‐hydroxykynurenine transaminase (PDB: 6MFB), with binding energy of −7.2 kcal/mol, indicating predicted complementarity with the HKT binding cavity. Squalene also showed the most favorable docking score for AChE (PDB: 6XYU), a central enzyme in insect cholinergic neurotransmission, with a binding energy of −10.2 kcal/mol (Table S7), making it a promising candidate for subsequent experimental investigation (Table S7). Interaction analyses revealed that this compound established hydrophobic interactions with Tyr73, Tyr324, Phe330, Phe371, Ser238, Gly151, Gly150, Thr154, His480, Gly481, Trp83, Tyr370, Glu80, Tyr374, and Tyr71, Asp375 (Figure 5A).

**FIGURE 5:**
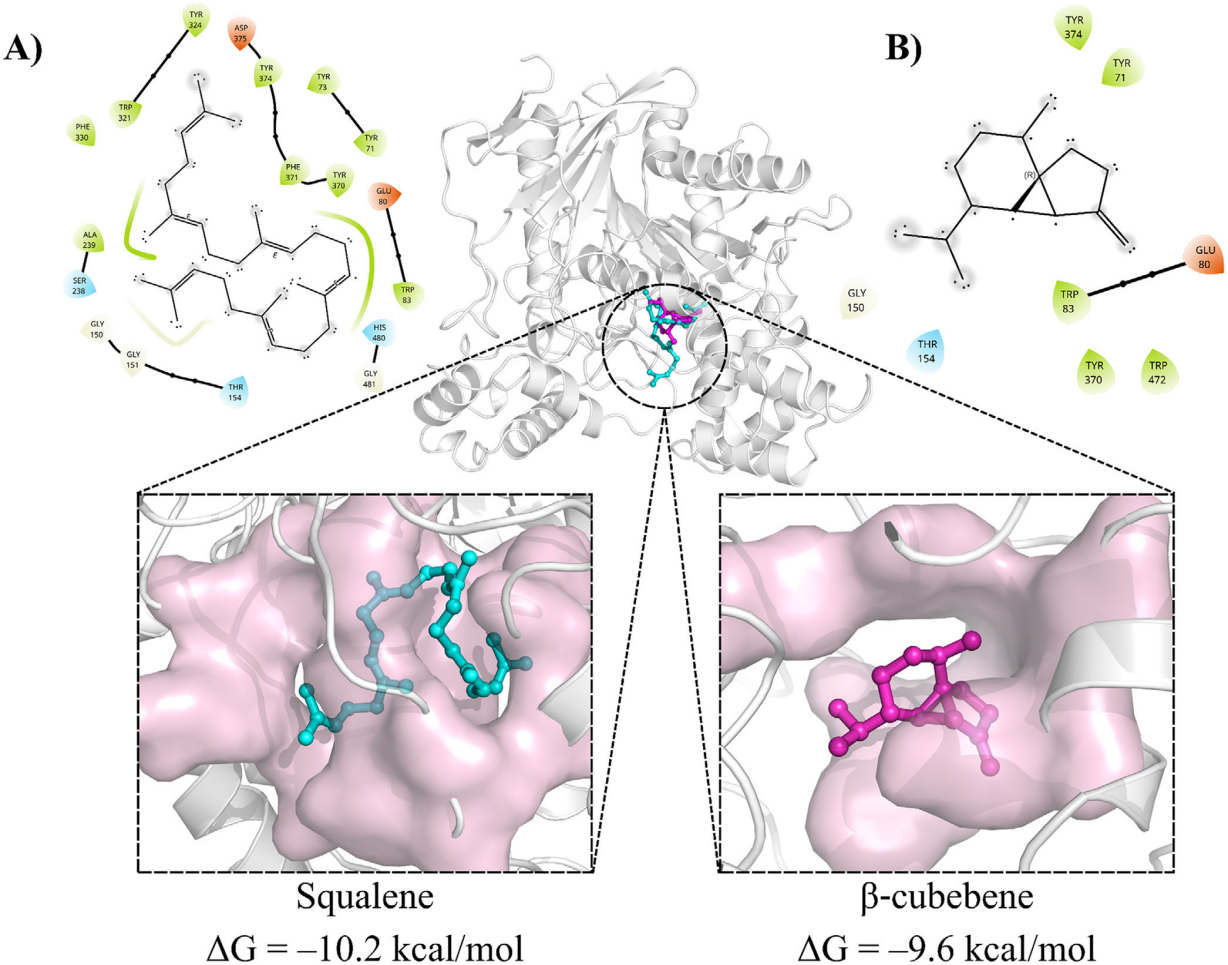
2D and 3D representations of molecular docking of the compounds from the supercritical extracts of *P. cattleianum* and *C. citriodora* most promising for interaction with the active site of *Drosophila melanogaster* acetylcholinesterase (DmAChE; PDB: 6XYU), used here as a homologous structural model for insect AChE in the absence of a resolved *Aedes aegypti* AChE crystal structure. (A) Squalene and (B) β‐cubebene. Hydrophobic interactions are shown in the light‐pink surface representation.

In the supercritical extract of *C. citriodora*, tetraprenol showed a favorable predicted interaction with SCP‐2 (PDB: 1PZ4), with binding energy of −8.7 kcal/mol, and the most favorable score against 3‐hydroxykynurenine transaminase (PDB: 6MFB), reaching −6.7 kcal/mol. These results suggest that tetraprenol can simultaneously interfere with the transport of essential lipids and tryptophan‐dependent metabolic pathways, two processes critical for larval development. In the same extract, β‐cubebene stood out for its strong interaction with AChE (PDB: 6XYU), with binding energy of −9.6 kcal/mol, indicating a favorable predicted interaction with the AChE model. Interaction analyses at the active site revealed that these compounds established hydrogen‐bond networks and specific hydrophobic contacts with catalytic and structural residues, contributing to the stabilization of protein–ligand complexes (Table S8) and (Figure 5B).

For the *P. guajava* compounds, similar affinities were observed for β‐sitosterol and squalene against sterol transporter protein‐2 (PDB: 1PZ4) and AChE (PDB: 6XYU), with binding energies of −9.8 and −10.2 kcal/mol, respectively. These results describe favorable predicted ligand–cavity complementarity for the evaluated targets but do not establish experimental binding or biological activity. For 3‐hydroxykynurenine transaminase (PDB: 6MFB), the best performance was observed for nerolidol, which presented binding energy of −7.0 kcal/mol, indicating a predicted interaction with HKT (Table S9) and (Figure 6).

**FIGURE 6:**
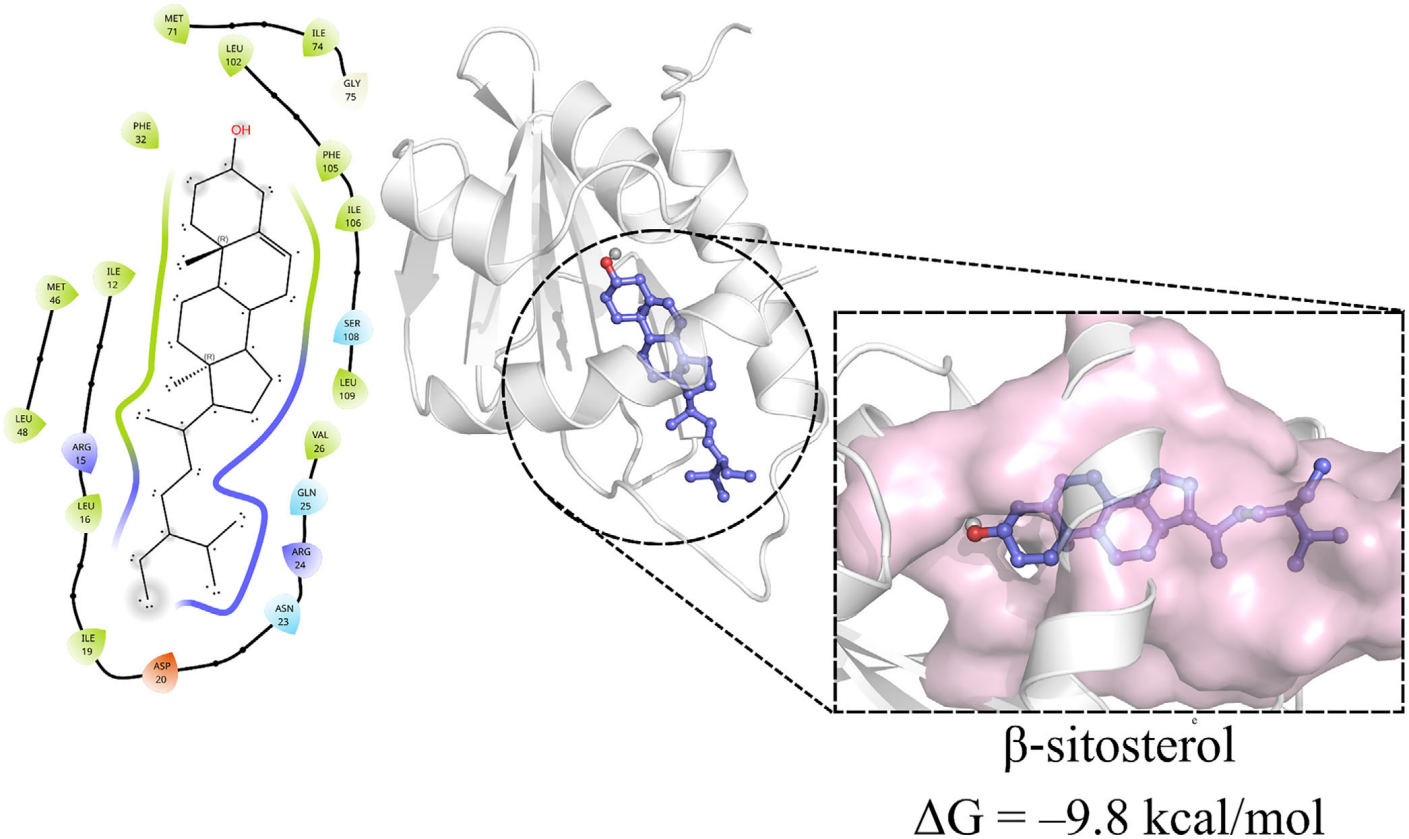
2D and 3D representations of the interaction of β‐sitosterol with the sterol‐2 transporter protein of *Aedes aegypti* (PDB: 1PZ4). The predominantly hydrophobic interactions are represented in the light pink surface representation.

In the supercritical extract of *P. cauliflora*, squalene showed favorable predicted interactions with key proteins involved in *A. aegypti* metabolism. This compound showed the most favorable binding scores against all three proteins evaluated: SCP‐2 (PDB: 1PZ4), 3‐hydroxykynurenine transaminase (PDB: 6MFB), and AChE (PDB: 6XYU), with energies of −9.4, −7.1, and −10.2 kcal/mol, respectively (Table S10).

For *E. uniflora*, the phytoconstituent 2‐[(2E,6Z)‐7‐(hydroxymethyl)‐3,11‐dimethyl‐dodeca‐2,6,10‐trienyl]‐1,4‐benzoquinone showed a favorable predicted interaction with SCP‐2 (PDB: 1PZ4), with binding energy of −9.9 kcal/mol, which showed a favorable predicted score for AeSCP‐2. In this same extract, squalene showed favorable predicted scores across more than one evaluated target, exhibiting the lowest binding energies against 3‐hydroxykynurenine transaminase (PDB: 6MFB) and AChE (PDB: 6XYU), with values of −7.1 and −10.2 kcal/mol, respectively (Table S11) and (Figure 7).

**FIGURE 7:**
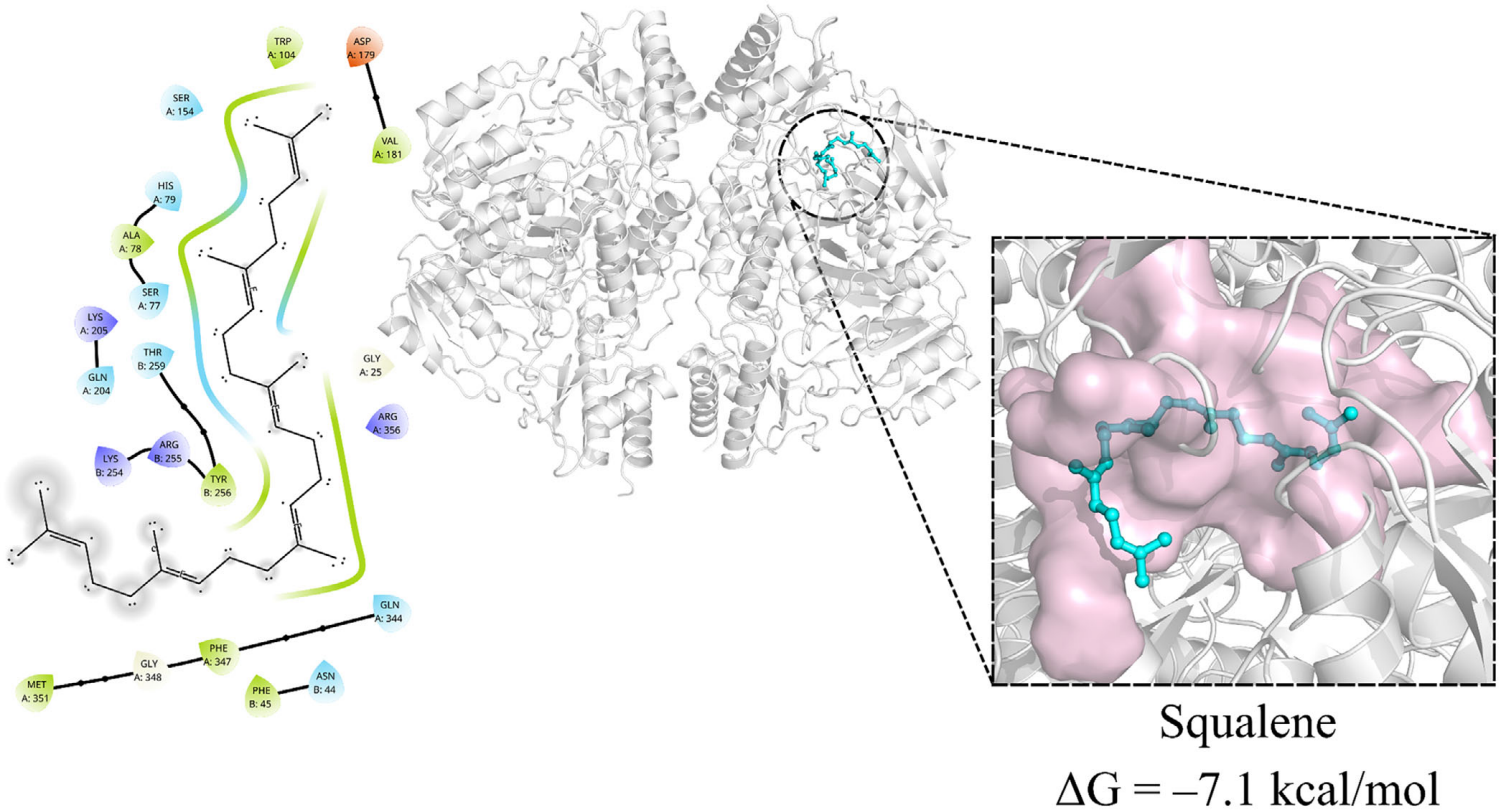
2D and 3D representations of the interaction of squalene with 3‐hydroxykynurenine transaminase from *A. aegypti* (PDB: 6MFB), highlighting the positioning of the ligand in the binding cavity and the predominance of hydrophobic interactions represented in the light pink colored surface analysis.

### 2.7 Molecular Dynamics and MM/PBSA Analyses

The AeSCP‐2–β‐sitosterol complex exhibited limited structural deviation during the 100 ns production trajectory (Figure S2A–C). The protein‐backbone RMSD averaged 1.74 ± 0.31 Å, while the ligand RMSD after protein fitting averaged 1.58 ± 0.37 Å. The mean Cα RMSF was 0.76 Å, with the largest fluctuations concentrated at the N‐ and C‐terminal regions, reaching 6.09 and 1.84 Å, respectively, whereas 85.0% of the Cα positions exhibited RMSF values below 1.0 Å. β‐Sitosterol remained largely shielded from the solvent, with a solvent‐accessible surface area of 0.524 ± 0.169 nm^2^ within the complex. The minimum protein–ligand distance averaged 0.197 ± 0.009 nm, and 569 ± 47 protein–ligand atom pairs were located within 0.40 nm. Hydrogen bonds were infrequent, averaging 0.048 ± 0.214 per frame, with at least one hydrogen bond detected in only 4.8% of the trajectory frames. Together, the limited ligand displacement, low solvent exposure, persistent close‐contact network, and sparse hydrogen bonding are consistent with a predominantly nonpolar interaction environment.

Across the 1001 snapshots, the MM/PBSA estimate was −7.08 ± 4.23 kcal·mol^−^^1^ (Figure S2D). Favorable gas‐phase interactions (−54.25 ± 2.93 kcal·mol^−^^1^) were dominated by the van der Waals contribution (−53.60 ± 2.78 kcal·mol^−^^1^), while the electrostatic contribution was small (−0.65 ± 1.71 kcal·mol^−^^1^). These interactions were largely offset by the total solvation contribution (+47.17 ± 3.27 kcal·mol^−^^1^). The polar‐solvation term was +17.14 ± 2.74 kcal·mol^−^^1^, whereas the inp = 2 nonpolar‐solvation model yielded cavity and dispersion contributions of −40.04 ± 1.33 and +70.06 ± 1.50 kcal·mol^−^^1^, respectively. The estimated −TΔS penalties were +7.44 ± 0.27 kcal·mol^−^^1^ using IE and +7.14 ± 0.29 kcal·mol^−^^1^ using C2. The corresponding entropy‐adjusted estimates were +0.36 ± 4.24 and +0.06 ± 4.24 kcal·mol^−^^1^, respectively. Thus, although β‐sitosterol remained structurally associated with AeSCP‐2, the favorable gas‐phase interactions were largely counterbalanced by solvation and approximate entropic contributions. These results should therefore be interpreted as model‐dependent comparative estimates rather than evidence of strongly favorable absolute binding.

## 3 Discussion

The present study demonstrates that supercritical extracts from five Myrtaceae species exhibit relevant but species‐dependent larvicidal activity against *A. aegypti*. The differences observed in magnitude, concentration dependence, and temporal progression indicate that larvicidal performance cannot be considered a uniform property of the botanical family or predicted solely from taxonomic proximity. *P. cattleianum* and *E. uniflora* showed particularly high activity within the tested range, whereas *P. cauliflora* exhibited a weaker and more heterogeneous response.

This variability is consistent with previous studies demonstrating substantial differences in chemical composition and larvicidal activity among Myrtaceae species and even among samples of the same species. Species‐dependent larvicidal activity has been reported [22] for essential oils from Myrtaceae, including *E. uniflora*, *P. cattleianum*, and *P. guajava*. Likewise, variation among *P. guajava* cultivars has been associated with marked differences in both volatile composition and larvicidal activity [23], while seasonal variation has been shown to modify the composition and biological activity of *P. cattleianum* oils [24]. More broadly, a meta‐analysis focused on Myrtaceae confirmed that seasonality significantly influences essential‐oil composition [25]. These observations reinforce the importance of interpreting larvicidal activity in relation to the specific chemical profile of each botanical preparation rather than as an invariant property of a species.

The findings for *E. uniflora* are supported by recent independent studies. Da Silva et al. [16]. demonstrated larvicidal activity of *E. uniflora* preparations and their major constituents against *A. aegypti*, while Aguiar et al. [26]. reported activity for both the essential oil and isolated curzerene. Curzerene was also abundant in the *E. uniflora* extract evaluated here. This convergence increases the biological plausibility of this chemical background but does not establish curzerene as the compound responsible for the activity observed in the present crude extract, since its individual contribution was not experimentally tested.

A similar caution applies to *C. citriodora*. Citronellal and citronellol predominated in its extract, and citronellal‐rich preparations of this species have previously shown larvicidal activity against *A. aegypti* [27]. However, differences in extraction method, geographic origin, composition, and experimental conditions preclude direct comparison of potency or attribution of activity to citronellal alone. Supercritical extraction also differs fundamentally from hydrodistillation in selectivity and recovery of volatile and less volatile constituents, making comparisons among preparations informative but not directly quantitative.

*Psidium cattleianum* merits separate consideration because previous studies indicate that both its volatile composition and larvicidal activity may vary substantially according to season and plant material [22, 24]. This variability reinforces that larvicidal potential should not be regarded as a fixed species‐level property, but rather as a characteristic of a particular botanical preparation with a defined chemical profile. This point is especially relevant when comparing the present supercritical extract with essential oils obtained by hydrodistillation, as these extraction approaches differ in selectivity and in the recovery of volatile and less volatile constituents. Accordingly, agreement between the present findings and previous reports on *P. cattleianum* should be interpreted qualitatively, rather than as evidence of directly comparable potency or of a shared active constituent. Although the extract showed a sesquiterpene‐rich profile, the covariance among its constituents prevents attribution of the observed activity to caryophyllene, guaia‐3,9‐diene, β‐cadinene, α‐selinene, or any other individual compound. Thus, *P. cattleianum* should be regarded as a priority candidate for fractionation and follow‐up bioassays, including tests with isolated constituents and defined mixtures.

More broadly, the LC_50_ estimates should be interpreted together with the observed concentration‐response patterns. For several highly active extracts, mortality already exceeded 50% at the lowest concentration tested, making the corresponding LC_50_ estimates extrapolative rather than fully bracketed by experimental observations. Conversely, the estimate for *P. cauliflora* was affected by high dispersion and a weakly monotonic response. Thus, the complete concentration‐response profile provides a more robust basis for comparative interpretation than isolated ranking by LC_50_.

### 3.1 Multivariate Chemical Structure and Mortality

The PCA confirmed that the extracts were strongly differentiated by chemical composition, with clear species‐ and concentration‐dependent trajectories. Importantly, many constituents exhibited similar loading patterns, indicating that the principal components represent covarying chemical assemblages rather than independent molecular effects. Mortality, projected as a supplementary variable, was not significantly associated with either of the first two components.

This does not indicate that composition is unrelated to larvicidal activity. Rather, it shows that the principal dimensions of chemical variation do not translate into a simple linear toxicity gradient. In chemically complex extracts, the compounds that explain the greatest compositional variance are not necessarily those most strongly associated with biological response. This distinction is consistent with the pronounced chemotypic and seasonal variability previously reported for Myrtaceae [23, 24, 25].

The regression tree provided a complementary nonlinear analysis and identified a limited set of chemical variables useful for partitioning mortality profiles. Nevertheless, its cross‐validated predictive performance was modest, and the selected variables should therefore be interpreted as markers of chemical configurations associated with different response patterns rather than as independent determinants of toxicity. This distinction is particularly important because the constituents covary within crude extracts. A compound with high variable importance may therefore represent a broader compositional profile rather than exerting an isolated biological effect.

For the same reason, the present dataset cannot distinguish additive, synergistic, or antagonistic interactions among individual constituents. Such claims require experiments with isolated compounds or defined mixtures and formal interaction analysis. Duarte et al. [28]. used a combination‐index approach to distinguish additive, synergistic, and antagonistic interactions among terpenoids. Accordingly, the present chemometric analyses are most useful for prioritizing compounds and compositional patterns for subsequent experimental testing, rather than assigning mechanism or causality.

### 3.2 Molecular Targets and Computational Prioritization

The molecular targets proposed in this study (AChE, SCP‐2, and 3‐hydroxykynurenine transaminase) were selected based on their established physiological relevance in insects and previous evidence supporting their potential as insecticidal targets. It is well established that docking scoring functions are not reliable predictors of true binding affinity, as they generally neglect solvent effects, hydrogen‐bonding accuracy, and the true conformational dynamics of the protein–ligand system [29]. Accordingly, docking results indicate predicted binding potential only; they do not account for bioavailability, membrane permeability, metabolic stability, or actual enzyme inhibition under physiological conditions, and therefore cannot, by themselves, confirm a mechanism of action or physiological pathway modulation. The multitarget mechanisms proposed here should accordingly be interpreted as testable hypotheses generated from the computational data, rather than as demonstrated biological effects, pending confirmation through enzymatic inhibition assays or other functional in vitro and in vivo approaches.

The squalene result also illustrates an important limitation of the present in silico analysis. Although it yielded favorable docking scores, squalene has not been tested here as an isolated larvicide, and its intracellular availability and actual engagement with the proposed targets remain unknown. Its high lipophilicity may further limit its dispersion in the aqueous larval environment and access to intracellular protein targets. Therefore, the docking results should not be interpreted as evidence that squalene is responsible for larvicidal activity; rather, they provide a rationale for subsequent biochemical, toxicological, and formulation studies.

To further assess one of these predicted interactions beyond the static docking model, the AeSCP‐2–β‐sitosterol complex was subjected to a 100 ns molecular dynamics (MD) simulation. β‐Sitosterol remained associated with the binding cavity, showing limited displacement, low solvent exposure, and sustained close contacts with the protein throughout the trajectory. The infrequent hydrogen bonds, together with the predominance of the gas‐phase van der Waals contribution, were consistent with stabilization mainly through nonpolar packing. Although solvation and approximate entropic contributions attenuated the energetic favorability estimated by MM/PBSA, retention of the ligand within the cavity reinforced the structural plausibility of the proposed binding mode under the simulated conditions. Collectively, the MD and MM/PBSA findings provide additional computational support for the AeSCP‐2–β‐sitosterol association and justify experimental investigation of its potential relevance to the larvicidal response, while direct conclusions regarding experimental binding affinity and AeSCP‐2 inhibition still require functional confirmation [30, 31].

The biological relevance of the selected targets is supported by evidence extending beyond computational studies. AeSCP‐2 participates in cholesterol transport and lipid homeostasis, and chemical inhibitors of mosquito SCP‐2 have been shown experimentally to interfere with cholesterol uptake and exert larvicidal activity [32, 33]. HKT contributes to the detoxification of 3‐hydroxykynurenine, and biochemical studies have demonstrated that mosquito HKT can be inhibited by small molecules, supporting its potential as a selective insecticidal target [15, 21]. AChE is essential for cholinergic neurotransmission, and inhibition of its activity has been experimentally observed in *A. aegypti* larvae exposed to larvicidal plant‐derived preparations [34]. These previous findings justify the biological selection of the three targets but do not imply that their inhibition occurred in larvae exposed to the extracts investigated here. Likewise, direct correspondence between regression‐tree importance and docking rank should not be expected because the chemometric analysis describes covarying crude‐extract profiles, whereas docking evaluates individual ligand–protein pairs.

Finally, the molecular docking findings identify promising isolated compounds for future investigation. The highest‐ranked ligands for SCP‐2, HKT, and AChE can be prioritized for synthesis or purification, followed by testing in biological models, individual larvicidal bioassays, and target‐specific functional assays. Similar strategies have already been employed to develop HKT‐targeted 1,2,4‐oxadiazole derivatives or essential oil‐based nanoemulsions optimized for interaction with AChE and SCP‐2 [28, 35].

### 3.3 Implications and Limitations

The docking results provide exploratory hypotheses regarding structurally plausible ligand–target interactions rather than direct evidence of the mechanisms underlying larval mortality. The three selected targets, SCP‐2, HKT, and AChE, are involved in physiologically relevant processes in mosquitoes, including sterol transport, 3‐hydroxykynurenine detoxification, and cholinergic neurotransmission, and have previous experimental or computational support as insecticidal targets [15, 18, 19, 20, 21, 32, 33, 34]. Accordingly, favorable docking scores can be used to prioritize compounds for subsequent investigation, but do not establish experimental binding, target inhibition, bioavailability, or contribution to the larvicidal effect.

The relationship between the docking and chemometric results should also be interpreted cautiously. PCA and regression‐tree analyses evaluated naturally covarying chemical profiles of crude extracts, whereas docking examined individual ligand–protein interactions. Therefore, a compound showing a favorable docking score would not necessarily be expected to emerge as an important predictor in the regression tree, and agreement between the two approaches does not establish causality. Likewise, because individual constituents were not experimentally manipulated, the present data cannot distinguish additive, synergistic, antagonistic, or independent effects. These possibilities require assays with isolated compounds and defined mixtures.

Nevertheless, the occurrence of favorable predicted interactions with proteins involved in distinct physiological processes is compatible with the broader multitarget framework proposed for botanical insecticides [36]. In the present study, this should be regarded as a hypothesis rather than a demonstrated mechanism. The computational analyses identify candidate ligand–target pairs that can now be prioritized for direct testing, following strategies previously applied to HKT‐directed compounds and other insecticidal candidates.

Taken together, the study supports a progression from demonstrated larvicidal activity to experimentally testable molecular hypotheses. Its main contribution lies in showing that chemically distinct Myrtaceae extracts produce different larvicidal profiles and that these differences can be investigated through complementary concentration‐response, chemometric, docking, and molecular‐dynamics approaches without assuming that a single constituent or target explains the entire response. This integrated strategy therefore provides a rational basis for selecting extracts, compounds, and molecular targets for subsequent experimental validation.

Regarding the limitations of this study, compound assignments were based on mass‐spectral and retention‐index evidence without confirmation using authentic standards. For some highly active extracts, the tested concentration range did not fully bracket 50% mortality, limiting the precision of LC_50_ estimates. Strong covariance among constituents prevents attribution of regression‐tree partitions to independent compound effects, while the modest cross‐validated performance indicates that the model is more suitable for hypothesis generation than broad prediction. Finally, docking, redocking, MD, and MM/PBSA remain computational approaches, and no direct inhibition of SCP‐2, HKT, or AChE was measured. Future studies should therefore prioritize lower concentrations for the most active extracts, isolated compounds and defined mixtures, direct target‐specific assays, and evaluation of formulation stability and nontarget effects.

## 4 Conclusions

The five Myrtaceae extracts exhibited species‐dependent larvicidal activity. The integrated analyses generated hypotheses regarding putative ligand–target associations and identified chemical profiles and candidate compounds for subsequent investigation. Differences in composition coincided with differences in mortality and docking scores, but the present data neither identify the constituents responsible for toxicity nor demonstrate a multitarget mechanism. In particular, the favorable docking scores obtained for squalene and tetraprenol should be interpreted only as a basis for direct experimental testing.

Overall, these findings support Myrtaceae extracts as sources of chemically diverse candidates for further larvicidal and target‐specific investigation. Future studies should evaluate lower concentrations of the most active extracts, isolated constituents and defined mixtures, direct target inhibition, environmental stability, formulation performance, and effects on nontarget organisms before field applications are considered.

### 4.1 Experimental Section

#### 4.1.1 Plant Material Preparation and Supercritical Fluid Extraction Process

The plant material (leaves) from five species of the Myrtaceae family (*E. uniflora, P. cauliflora, C. citriodora, P. guajava*, and *P. cattleianum*) were collected during the pre‐flowering stage in the municipality of Frederico Westphalen, near the campus of the Federal University of Santa Maria, Rio Grande do Sul, Brazil, in November 2024. The leaves were collected in the early morning, dried in a forced air circulation oven at 40°C for 7 days, until the mass stabilized, and then ground in a Willey knife mill. with the particle size standardization in 30 mesh [37]. Botanical identification was confirmed by a specialist, and a voucher specimen of each species was deposited in a herbaria of the Federal University of Santa Maria (SMDB 22272, SMDB 22271, SMDB 22276, and SMDB 22269) and at the Community University of the Chapecó Region—UNO Chapecó (scientific collection–5102).

Supercritical extraction was performed at 40°C and 150 bar using an experimental apparatus. The Singularity supercritical fluid extraction system consisted of a solvent reservoir, two thermostatic baths, a preparative piston pump (Jasco, model PU‐4587), a 100 mL jacketed extraction vessel, an absolute pressure transducer equipped with a portable programmer, a collection container with a glass tube and a cold trap [38, 39]. Portions of 30 g (±0.05 g) of the dehydrated plant materials were loaded into the extraction container. The solvent was pumped at a constant flow rate of 2 mL.min‐1 through the extraction bed, which was supported by two 300 mesh wire discs at both ends, and maintained in contact with the plant matrix for at least 30 min to allow system stabilization.

#### 4.1.2 Chemical Characterization of Extracts and Quantification of Compounds

The chemical profile of the extracts of *C. citriodora, E. uniflora, P. cattleianum, P. cauliflora, and P. guajava* was determined using an Agilent 7890B gas chromatograph coupled to 5977A MSD mass spectrometry. The experimental conditions followed those described by Radunz et al. [39]. Samples were dissolved in dichloromethane (extract: 20 mg/5 mL) and injected (1 µL) in split mode (20:1). Chromatographic separation was performed using an Agilent 19091S column (30 m × 250 µm × 0.25 µm) with helium as the carrier gas at 1.0 mL/min.

The oven temperature was programmed from 60°C to 300°C at a rate of 10°C min^−^^1^. The temperatures of the injector, transfer line, and ion source were set to 280°C, 150°C, and 230°C, respectively. Electron ionization (IE) was performed at 70 eV with mass scanning of 40 to 400 *m*/*z*. Compound assignments were based on the combined comparison of electron‐ionization mass spectra against the NIST 5.01 library and experimentally determined linear retention indices (LRIs). LRIs were calculated using a homologous series of C_7_–C_30_ n‐alkanes according to the Van den Dool and Kratz equation and compared with literature values obtained under comparable chromatographic conditions. The NIST library match factor, experimental LRI, literature LRI, and the difference between experimental and literature values were recorded for each compound. Because authentic reference standards were not analyzed, the compounds were considered putatively identified rather than unequivocally confirmed [40].

### 4.2 Larval Colony

The larvae of *A. aegypti* were obtained from the Ecological Entomology Laboratory (LABENT‐Eco) of the Community University of the Chapecó Region (Unochapecó). The eggs were hatched in 1 liter of distilled water. The larvae were maintained at a density of 500–750 larvae/L and fed with Spirulina fish feed (Alcon, Camboriú, SC, Brazil) [41].

### 4.3 Larvicidal Bioassay With *Aedes aegypti*

Larvicidal activity was evaluated following World Health Organization guidance [42], with the experimental adaptations described below. Supercritical leaf extracts of *C. citriodora, E. uniflora, P. cauliflora, P. cattleianum*, and *P. guajava* were dissolved in acetone and tested at nominal concentrations of 60, 120, 180, 240, and 300 µg mL^−^^1^. A solvent control containing 0.4% acetone without plant extract was tested concurrently. A positive‐control concentration–response assay was performed using the commercial larvicide VectoBac WG (Bacillus thuringiensis var. israelensis, Bti) under the same experimental conditions used for the plant extracts. No concentration categories were used in the statistical analyses; each nominal concentration was retained at its measured experimental level.

Each extract‐concentration treatment and the solvent control were represented by six physically independent 50‐mL microcosms, each containing 15 third‐instar larvae. The microcosm was therefore the experimental unit; larvae within a microcosm were subsamples. The six microcosms assigned to each treatment were prepared on the same day and monitored concurrently throughout the 72‐h exposure period, representing independent experimental units within the same experimental run rather than separate experiments conducted on different days. Larvae were not fed during the 72‐h exposure period. Abbott's correction was not applied because no mortality occurred in the concurrently tested solvent‐control group. Cumulative mortality was recorded at 0, 1, 2.5, 5, 10, 24, 48, and 72 h. Larvae were classified as dead when no spontaneous movement was observed and no response occurred after gentle probing with fine forceps. Bioassays were conducted at 27 +/−2 degrees C, 70%–80% relative humidity, and a 12:12 h light:dark photoperiod. The primary endpoint for concentration‐response modelling, PCA projection, and regression‐tree analysis was cumulative mortality at 72 h. The previously calculated arithmetic mean across observation times was not used because it averages cumulative responses over unequal time intervals and is not a final‐mortality endpoint.

### 4.4 Concentration‐Response Analysis

For each species, concentration‐response relationships were fitted to replicate‐level dead/alive counts at 72 h using generalized linear models with a probit link and a quasibinomial variance function. The quasibinomial specification accommodated extra‐binomial variation among independent microcosms. Concentration was log10‐transformed for model fitting. The median lethal concentration (LC50) was obtained from the fitted intercept and slope, and 95% confidence intervals were calculated on the log10‐concentration scale using the delta method and back‐transformed to the original scale. We report all replicate observations, concentration‐specific mean mortality +/−SD, fitted curves, and 95% confidence bands. LC50 estimates were flagged when the observed concentrations did not bracket 50% mortality, when the estimated LC50 lay outside the tested range, or when the mean concentration‐response pattern was weak or non‐monotonic. These flags were used to distinguish interpolation supported by the experimental range from extrapolation or unstable estimation [43].

### 4.5 Chemical‐Profile Quality Control

Chemical variables were parsed using a decimal‐aware routine before analysis. Values were audited within each nominal treatment because the six microcosms shared the same treatment‐level chemical profile. For treatment‐chemical combinations with discrepant replicate rows, the within‐treatment median was used to construct the multivariate and tree‐model predictor matrices, thereby limiting the influence of isolated data‐entry discrepancies. Six discrepancies involving (R)‐(+)‐citronellic acid and β‐sitosterol in CC240, CC300, and EU60 were retained in a quality‐control table for verification against the original analytical records. All mortality columns, including the previous composite mortality column and the eight time‐specific mortality variables, were excluded from the chemical predictor matrix before model fitting.

### 4.6 In Silico Studies

The three‐dimensional structures of the chemical constituents identified in the supercritical extracts were obtained from the PubChem database in SDF format [44]. The stereochemical assignments provided by PubChem were retained during processing. Protonation states at pH 7.4 were assigned using Open Babel 3.1.1 [45]. Explicit hydrogen atoms were added, and up to 100 conformers per compound were generated using the ETKDGv3 method implemented in RDKit 2025.09.5 [46], with a fixed random seed of 1234. The conformers were minimized using the MMFF94 force field [47] for up to 500 iterations, with UFF used when MMFF94 parameters were unavailable [48]. Non‐converged conformers were discarded, and the lowest‐energy converged conformer was selected. The ligands were subsequently prepared using Meeko 0.7.1 and exported in PDBQT format [49].

The crystallographic structures of *A. aegypti* SCP‐2 (AeSCP‐2; PDB: 1PZ4), *A. aegypti* 3‐hydroxykynurenine transaminase (AeHKT; PDB: 6MFB), and *D. melanogaster* acetylcholinesterase (AChE; PDB: 6XYU) were obtained from the Protein Data Bank [50]. These targets were selected because of their respective roles in lipid transport and mosquito development, kynurenine metabolism and 3‐hydroxykynurenine detoxification, and cholinergic neurotransmission. The *D. melanogaster* AChE structure was used as an experimentally determined homologous model of insect AChE. Water molecules, ions, glycans, co‐crystallized ligands, and other nonprotein heteroatoms were removed. Hydrogen atoms and protonation states of titratable residues at pH 7.4 were assigned using PROPKA as implemented in PDB2PQR 3.7.1 [51]. The receptors were then prepared using Meeko 0.7.1 and exported as rigid structures in PDBQT format [49].

The binding cavities were defined from the positions of the corresponding co‐crystallized ligands. Molecular docking was performed using AutoDock Vina 1.2.7 [52, 53], with rigid receptors and flexible ligands. Cubic search boxes of 20 × 20 × 20 Å were centered at *x* = 25.18, *y* = 29.74, and *z* = 54.91 Å for 1PZ4; *x* = 55.69, *y* = 81.43, and *z* = 70.94 Å for 6MFB; and *x* = 33.12, *y* = 67.65, and *z* = 9.89 Å for 6XYU. One docking run was performed for each ligand–receptor pair using a fixed seed of 1234, an exhaustiveness value of 90, a maximum of 20 output poses, and an energy range of 20 kcal/mol. All remaining parameters were maintained at their default values. The top‐ranked pose, defined by the most favorable Vina score, was selected for interaction analysis and visualized using PyMOL 2.4.0. The corresponding co‐crystallized molecules were subsequently used as structural references for redocking validation.

Redocking was performed to evaluate the ability of the docking protocol to reproduce the experimentally observed ligand poses. Heavy‐atom RMSD values between the redocked poses and the corresponding crystallographic conformations were calculated using DockRMSD 1.1 [54]. For each target, the lowest RMSD among the 20 generated poses was considered for protocol validation. An RMSD below 2.0 Å was considered indicative of satisfactory pose reproduction. The lowest RMSD values were 1.859 Å for AeSCP‐2 (PDB: 1PZ4; pose 15), 0.543 Å for AeHKT (PDB: 6MFB; pose 13), and 0.428 Å for AChE (PDB: 6XYU; pose 5). As all RMSD values were below the established threshold, the docking protocol was considered capable of reproducing the crystallographic ligand orientations for the three targets [55]. The molecular affinity parameters and interacting residues obtained for the co‐crystallized control ligands I40 and PLM are provided in Table S12.

Molecular dynamics (MD) simulation was performed for the AeSCP‐2–β‐sitosterol complex (PDB: 1PZ4) using the top‐ranked docking pose as the initial configuration. The soluble protein–ligand complex was prepared using CHARMM‐GUI [56], and GROMACS‐compatible input files were generated. The protein was described using the CHARMM36 force field [57], while β‐sitosterol was represented using the CHARMM SITO parameters included in the generated topology. The complex was solvated with TIP3P water [58] in a cubic box of approximately 65 × 65 × 65 Å, neutralized, and ionized with NaCl to a final concentration of 0.15 M, resulting in a system containing 25 699 atoms. After energy minimization, the system was equilibrated for 125 ps under the canonical ensemble (NVT), followed by 1 ns under the isothermal–isobaric ensemble (NPT). A 100 ns production simulation was subsequently performed at 303.15 K and 1 bar under periodic boundary conditions using GROMACS 2026.3 [59]. a 2 fs integration step was used, and coordinates were saved every 0.1 ns. After correction of periodic‐boundary artifacts and fitting to the protein backbone, 1001 frames were analyzed for protein‐backbone RMSD, protein Cα RMSF, ligand RMSD after protein fitting, ligand solvent‐accessible surface area, minimum protein‐ligand distance, contacts within 0.40 nm, and protein–ligand hydrogen bonds. hydrogen bonds were defined by a donor–acceptor distance of ≤0.35 nm and a donor‐hydrogen‐acceptor angle of ≥ 150°. MM/PBSA calculations were performed using gmx_MMPBSA v1.6.5. and the single‐trajectory approach, considering all 1001 snapshots sampled every 0.1 ns throughout the 100 ns trajectory [60].

Nonpolar solvation was treated using the inp = 2 model, which separately accounts for cavity and dispersion contributions [61]. The binding‐energy estimate comprised van der Waals, electrostatic, polar‐solvation, cavity, and dispersion terms. Entropic penalties were approximated at 303.15 K using both interaction entropy (IE) and the second‐order cumulant (C2) method, based on fluctuations in the gas‐phase protein–ligand interaction energy [62]. The entropy‐adjusted values were interpreted as comparative end‐point estimates rather than absolute experimental binding free energies [30, 31].

### 4.7 Statistical Analyses

Principal component analysis (PCA) was conducted on treatment‐level chemical composition to characterize the dominant multivariate gradients among extracts and concentrations [63]. Replicate rows were summarized to one centroid for each of the 25 extract‐concentration treatments. The six solvent‐control microcosms were represented by a single control centroid, with all chemical variables set to zero, yielding 26 treatment centroids. All 71 nonconstant chemical variables were centered and scaled to unit variance and entered as active variables in the PCA. Mortality at 72 h was excluded from axis construction and projected post hoc as a supplementary quantitative variable by correlating treatment‐level mean mortality with the PCA scores [64]. Consequently, the supplementary mortality vector describes association with the chemical ordination but cannot influence the components or establish chemical causation.

Associations between chemical composition and 72‐h larval mortality were examined using an analysis‐of‐variance regression tree implemented in rpart. The analysis included 150 independent microcosms nested within 25 extract‐concentration treatments (six microcosms per treatment; 15 larvae per microcosm; 2250 larvae in total). The solvent control was excluded because it contained no extract. All 71 nonconstant chemical variables were eligible predictors. A maximal tree was fitted with a minimum split size of 12 observations, a minimum terminal‐node size of six, a maximum depth of four, no surrogate splits, and an initial complexity parameter (CP) of zero.

Predictive performance and pruning were evaluated using 100 repeated, stratified, grouped fivefold cross‐validations. Grouping was performed at the extract‐concentration treatment level so that all six microcosms sharing a chemical profile occurred exclusively in either the calibration or validation subset. Within every repeat, each validation fold contained one concentration from each plant species. This design prevented identical treatment‐level chemical profiles from appearing simultaneously in training and validation data, which would underestimate prediction error in hierarchically structured observations [65, 66]. Candidate subtrees were generated by cost‐complexity pruning. The final CP was selected using the one‐standard‐error rule: we retained the largest CP whose mean validation RMSE was no more than one standard error above the minimum, favoring parsimony. Predictive performance was summarized from held‐out predictions using cross‐validated R‐squared, root mean squared error (RMSE), and mean absolute error (MAE); uncertainty was expressed as the SD and 2.fifth–97.fifth percentile interval across the 100 repeats. Variable importance was reported descriptively for the final tree. Tree split values were interpreted as sample‐dependent predictive partitions rather than causal or physiological thresholds [67]. All statistical procedures were performed in R version 4.4.1.

## Author Contributions

Conceptualization: U.A.B., R.C.P., G.D.C., J.D.M., and V.S.C. Methodology: U.A.B., R.C.P., G.D.C., J.D.M., and V.S.C. Validation: U.A.B., R.S.R., W.A.R.J., J.D.M., and G.D.C. Formal analysis: R.S.R., W.A.R.J., J.D.M., M.V.B., and I.R.G. Investigation: U.A.B., G.D.C., and R.C.P. Resources: V.S.C., J.D.M., and R.S.R. Data curation: U.A.B. and G.D.C. Writing – original draft: U.A.B. Writing – review & editing: U.A.B., G.D.C., W.A.R.J., J.D.M., V.S.C., R.S.R., M.V.B., and I.R.G. Visualization: U.A.B., R.S.R., W,A.R.J., M.V.B., and I.R.G. Supervision: V.S.C., J.D.M., R.S.R., and W.A.R.J. Project administration: V.S.C. and J.D.M. Funding acquisition: V.S.C. and J.D.M. All authors have read and agreed to the final version of the manuscript .

## Ethics Statement

Ethical approval was not required for this study because it involved only *A. aegypti* larvae, an invertebrate species. Under Brazilian Law No. 11,794/2008, review by an institutional animal ethics committee applies to animals belonging to the phylum Chordata, subphylum Vertebrata. All authors made substantial intellectual contributions to the conception, development, data analysis, and writing of the manuscript and approved the final version. The work is free from any form of plagiarism, self‐plagiarism, or fraudulent data. All sources have been properly cited and acknowledged.

## Conflicts of Interest

The authors declare no conflicts of interest.

## Supporting information



Supporting information for this article is available on the Harvard Dataverse under https://doi.org/10.7910/DVN/THGLJF.
**Supporting File 1**: cbdv71786‐supp‐0001‐SuppMat.docx.

**Supporting File 2**: cbdv71786‐supp‐0002‐Supplement statistical.xlsx.

## Data Availability

The data that support the findings of this study are openly available in Harvard Dataverse at https://doi.org/10.7910/DVN/THGLJF.
