# Supplementary material for: Integrated Experimental, Chemometric, and In Silico Analyses of Larvicidal Supercritical Extracts From Myrtaceae Species Against Aedes aegypti
Source: Chem Biodivers. 2026 Sep 30;23(10):e71786. doi: 10.1002/cbdv.71786 (PMC13626261; doi:10.1002/cbdv.71786)
Supplement: Supplementary file 1 [file CBDV-23-e71786-s001.docx]

**SUPPLEMENTARY MATERIAL**

**Table S1.** Raw cumulative mortality data for Aedes aegypti larvae exposed to Myrtaceae extracts. The table provides one row for each microcosm and observation time, including treatment identity, plant species, nominal extract concentration, replicate number, exposure time, and observed mortality percentage. Each treatment comprised six independent 50-mL microcosms containing 15 larvae each. Dead and surviving larval counts were reconstructed from the reported mortality percentages using the initial number of 15 larvae per microcosm; reconstruction-error columns are provided as internal consistency checks. The solvent control contained 0.4% acetone and no extract.

**Table S2.** Descriptive statistics for cumulative mortality of Aedes aegypti larvae across concentrations and exposure times. Mean mortality, SD, minimum, and maximum are reported for each treatment-by-time combination. Statistics were calculated from six independent microcosms, corresponding to 90 larvae per treatment. Measurements were obtained at 0, 1, 2.5, 5, 10, 24, 48, and 72 h. The table includes the five extract concentrations and the 0.4% acetone solvent control.

**Table S3.** Nominal concentrations of Myrtaceae extracts tested against Aedes aegypti larvae. For each plant species, the table reports the complete set of tested concentrations (60, 120, 180, 240, and 300 µg mL⁻¹), the number of independent microcosms per concentration, the number of larvae per microcosm, and the total number of larvae evaluated. Concentration was analyzed as a continuous quantitative variable; qualitative categories such as low, intermediate, or high dose were not used. The solvent control consisted of 0.4% acetone without extract and was treated separately from the positive concentrations.

**Table S4.** Quasibinomial probit estimates of the median lethal concentration of Myrtaceae extracts against Aedes aegypti larvae after 72 h. LC₅₀ values and their 95% confidence intervals are expressed in µg mL⁻¹ and were estimated separately for each plant species using quasibinomial generalized linear models with a probit link and log10-transformed concentration. Models included the five positive concentrations, with six independent microcosms and 15 larvae per microcosm at each concentration. The table also reports the fitted slope, dispersion parameter, Spearman correlation between concentration and mean mortality, whether observed mean mortality values bracketed 50%, and whether the estimated LC₅₀ occurred within the tested concentration range. The solvent control was excluded from model fitting because log10(0) is undefined. Estimates requiring extrapolation or lacking an estimable confidence interval should be interpreted cautiously.

**Table S5.** Selection of the regression-tree complexity parameter by repeated grouped cross-validation. Candidate complexity-parameter values were evaluated using 100 repetitions of grouped five-fold cross-validation. All six microcosms belonging to the same species-by-concentration treatment were kept within the same fold. For each candidate CP, the table reports mean R², RMSE, and MAE across repetitions, together with their SDs and percentile intervals. The most parsimonious tree whose RMSE was within one standard error of the minimum cross-validated RMSE was selected according to the one-standard-error rule.

**Table S6.** Cross-validated predictive performance of the selected regression tree. Performance was assessed using 100 repetitions of grouped five-fold cross-validation applied to 150 microcosms nested within 25 species-by-concentration treatment profiles. Reported statistics are the mean R², RMSE, and MAE across repetitions, with SDs and percentile-based 95% intervals. RMSE and MAE are expressed in mortality percentage points. The selected tree used CP = 0.002317 and yielded R² = 0.125, RMSE = 21.07 percentage points, and MAE = 12.38 percentage points. These metrics describe out-of-fold predictive performance and should not be interpreted as causal evidence for individual chemical compounds.

**Table S7.** Molecular affinity parameters between the best compounds of *P. cattleianum* against the proteins 6XYU, 1PZ4, 6MFB.

| **Complex**  **(binder-protein)** | **ΔG**  **(kcal/mol)** | **Amino acids that interact through hydrogen bonds** | **Amino acids that carry out hydrophobic interactions** |
| --- | --- | --- | --- |
| Squalene_6XYU  Squalene_1PZ4  B-bisabolene_6MFB | – 10.2  – 9.4  – 7.2 | –  –  – | Tyr73(A); Tyr324(A); PHE330(A); PHE371(A); SER238(A); GLY151(A); Gly150(A); Thr154(A); HIS480(A); Gly481(A); Trp83(A); Tyr370(A); Glu80(A); Tyr374(A); Tyr71(A); ASP375(A)  Ala81(P); Ile106(P); Ile12(P); Leu109(P); Phe32(P); Val26(P); Arg15(P); Asp20(P); Asn23(P); Gln25(P); Phe105(P); Leu48(P); Arg24(P); Met46(P); Ile74(P); Leu102(P); Leu64(P); Ile99(P)  SER154(A); Gly25(A); Asn44(B); Trp104(A); Gln344(A); Tyr256(B); Phe45(B); Met351(A); Phe347(A); ARG356(A); Lys205(A); |

**Table S8.** Molecular affinity parameters between the best compounds of *C. citriodora* against 6XYU, 1PZ4, 6MFB proteins.

| **Complex**  **(binder-protein)** | **ΔG**  **(kcal/mol)** | **Amino acids that interact through hydrogen bonds** | **Amino acids that carry out hydrophobic interactions** |
| --- | --- | --- | --- |
| B-cubebene_6XYU  tetraprenol_1PZ4  tetraprenol_6MFB | – 9.6  – 8.7  – 6.7 | -  Val26(P); Gln25(P); ARG24(P)  GLN344(A); Ser43(B); ASN44(B) | Glu80(A); Trp83(A); Tyr370(A); Thr154(A); Tyr71(A); Tyr374(A)  Leu102(P); Ile99(P); Glu103(P); Leu64(P); Met71(P); Ile106(P); Met46(P); Leu109(P); Ile12(P); Arg15(P); Leu48(P); Phe105(P); Ile19(P)  Gly25(A); Pro26(A); Gly346(A); Met351(A); Phe45(B); Arg356(A); Ile103(A); Tyr256(B); Phe347(A); Arg255(B); Lys205(A) |

**Table S9.** Molecular affinity parameters between the best compounds of *P. guajava* against 6XYU, 1PZ4, and 6MFB proteins.

| **Complex**  **(binder-protein)** | **ΔG**  **(kcal/mol)** | **Amino acids that interact through hydrogen bonds** | **Amino acids that carry out hydrophobic interactions** |
| --- | --- | --- | --- |
| Squalene_6XYU  β‑sitosterol_1PZ4  Squalene_1PZ4  Nerolidol_6MFB | – 10.2  – 9.8  – 9.4  – 7.0 | –  –  –  Gln344(A) | Tyr73(A); Tyr324(A); PHE330(A); PHE371(A); SER238(A); GLY151(A); Gly150(A); Thr154(A); HIS480(A); Gly481(A); Trp83(A); Tyr370(A); Glu80(A); Tyr374(A); Tyr71(A); ASP375(A)  Ser108(P); Ile12(P); Ile106(P); Leu109(P); Leu102(P); Arg24(P); Phe105(P); Val26(P); Ile19(P); Asp20(P); Asn23(P); Gln25(P); Arg15(P); Leu16(P); Leu48(P); Met46(P); Phe32(P);  Ala81(P); Ile106(P); Ile12(P); Leu109(P); Phe32(P); Val26(P); Arg15(P); Asp20(P); Asn23(P); Gln25(P); Phe105(P); Leu48(P); Arg24(P); Met46(P); Ile74(P); Leu102(P); Leu64(P); Ile99(P)  SER154(A); Pro24(A); Lys205(A); ARG356(A); Phe347(A); Tyr256(B); Phe45(B); ARG255(B); Lys254(B); ASN44(B) |

**Table S10.** Molecular affinity parameters between the best compounds of *P. cauliflora* against 6XYU, 1PZ4, 6MFB proteins.

| **Complex**  **(binder-protein)** | **ΔG**  **(kcal/mol)** | **Amino acids that interact through hydrogen bonds** | **Amino acids that carry out hydrophobic interactions** |
| --- | --- | --- | --- |
| Squalene_6XYU  Squalene_1PZ4  Squalene_6MFB | – 10.2  – 9.4  – 7.1 | –  –  – | Tyr73(A); Tyr324(A); PHE330(A); PHE371(A); SER238(A); GLY151(A); Gly150(A); Thr154(A); HIS480(A); Gly481(A); Trp83(A); Tyr370(A); Glu80(A); Tyr374(A); Tyr71(A); ASP375(A)  Ala81(P); Ile106(P); Ile12(P); Leu109(P); Phe32(P); Val26(P); Arg15(P); Asp20(P); Asn23(P); Gln25(P); Phe105(P); Leu48(P); Arg24(P); Met46(P); Ile74(P); Leu102(P); Leu64(P); Ile99(P)  Phe347(A); Tyr256(B); ASN44(B); Thr259(B); GLN204(A); Lys205(A); His79(A); Ala. 78(A); ASP179(A); Ser154(A); Val181(A); Trp104(A); Met351(A); Arg356(A); GLN344(A); Phe45(B); Arg255(B) |

**Table S11.** Molecular affinity parameters between the best compounds of *E. uniflora* against 6XYU, 1PZ4, 6MFB proteins.

| **Complex**  **(binder-protein)** | **ΔG**  **(kcal/mol)** | **Amino acids that interact through hydrogen bonds** | **Amino acids that carry out hydrophobic interactions** |
| --- | --- | --- | --- |
| Squalene_6XYU  2‑[(2E,6Z)-7-(hydroxymethyl)-3,11‑dimethyl‑dodeca‑2,6,10‑trienyl]‑1,4‑benzoquinone _1PZ4  Squalene_6MFB | – 10.2  – 9.9  – 7.1 | –  Arg15(P); Gln25(P); Val26(P); ARG24(P)  – | Tyr73(A); Tyr324(A); PHE330(A); PHE371(A); SER238(A); GLY151(A); Gly150(A); Thr154(A); HIS480(A); Gly481(A); Trp83(A); Tyr370(A); Glu80(A); Tyr374(A); Tyr71(A); ASP375(A)  Leu48(P); Phe105(P); Met46(P); Phe32(P); Met71(P); Ile106(P); Leu102(P); Leu109(P); Ile12(P); Ile19(P); Leu16(P); Asp20(P); Asn23(P)  Phe347(A); Tyr256(B); ASN44(B); Thr259(B); GLN204(A); Lys205(A); His79(A); Ala. 78(A); ASP179(A); Ser154(A); Val181(A); Trp104(A); Met351(A); Arg356(A); GLN344(A); Phe45(B); Arg255(B) |

**Table S12.** Molecular affinity parameters and interacting amino acids obtained from redocking the co-crystallized ligands I40 [9-(3-iodobenzylamino)-1,2,3,4-tetrahydroacridine] and PLM (palmitic acid) in the 6XYU and 1PZ4 protein structures, respectively.

| **Complex**  **(binder-protein)** | **ΔG**  **(kcal/mol)** | **Amino acids that interact through hydrogen bonds** | **Amino acids that carry out hydrophobic interactions** |
| --- | --- | --- | --- |
| I40_6XYU | – 12.5 | – | Tyr71(A); Trp83(A); Tyr162(A); Tyr324(A); Phe330(A); Tyr370(A); Phe371(A); Tyr374(A); Trp472(A); Thr154(A); His480(A); Glu237(A); Gly149(A); Gly150(A); Gly481(A) |
| PLM_1PZ4 | – 7.3 | Arg24(P); Gln25(P); Val26(P) | Ile12(P); Leu16(P); Ile19(P); Met46(P); Leu48(P); Leu102(P); Phe105(P); Ile106(P); Leu109(P); Arg15(P); Asp20(P) |

**Figure S1.** Time-dependent cumulative mortality of Aedes aegypti larvae exposed to Myrtaceae extracts. Lines connect mean cumulative mortality values measured at 0, 1, 2.5, 5, 10, 24, 48, and 72 h, and error bars represent SD among six independent microcosms. Each microcosm contained 15 larvae. Colors indicate the nominal extract concentrations of 60, 120, 180, 240, and 300 µg mL⁻¹. Panels correspond to the five plant species. The 0.4% acetone solvent control is not displayed in this figure but is reported in Tables S1 and S2.

**Figure S2.** Molecular dynamics and MM/PBSA analysis of the AeSCP-2–β-sitosterol complex over 100 ns. (A) Protein-backbone and protein-fitted β-sitosterol RMSD. (B) β-Sitosterol solvent-accessible surface area. (C) Protein Cα RMSF. (D) MM/PBSA energy decomposition. In A and B, light and solid traces represent per-frame values and 11 ns moving averages, respectively; dashed lines indicate means in A–C, and error bars represent SD in D.
